# Big behavior: challenges and opportunities in a new era of deep behavior profiling

**DOI:** 10.1038/s41386-020-0751-7

**Published:** 2020-06-29

**Authors:** Lukas von Ziegler, Oliver Sturman, Johannes Bohacek

**Affiliations:** 1 Department of Health Sciences and Technology, ETH, Laboratory of Molecular and Behavioral Neuroscience, Institute for Neuroscience, Zurich, Switzerland; 2 Neuroscience Center Zurich, ETH Zurich and University of Zurich, Zurich, Switzerland

**Keywords:** Stress and resilience, Behavioural methods

## Abstract

The assessment of rodent behavior forms a cornerstone of preclinical assessment in neuroscience research. Nonetheless, the true and almost limitless potential of behavioral analysis has been inaccessible to scientists until very recently. Now, in the age of machine vision and deep learning, it is possible to extract and quantify almost infinite numbers of behavioral variables, to break behaviors down into subcategories and even into small behavioral units, syllables or motifs. However, the rapidly growing field of behavioral neuroethology is experiencing birthing pains. The community has not yet consolidated its methods, and new algorithms transfer poorly between labs. Benchmarking experiments as well as the large, well-annotated behavior datasets required are missing. Meanwhile, big data problems have started arising and we currently lack platforms for sharing large datasets—akin to sequencing repositories in genomics. Additionally, the average behavioral research lab does not have access to the latest tools to extract and analyze behavior, as their implementation requires advanced computational skills. Even so, the field is brimming with excitement and boundless opportunity. This review aims to highlight the potential of recent developments in the field of behavioral analysis, whilst trying to guide a consensus on practical issues concerning data collection and data sharing.

## Introduction

### Measuring behavior—the past

In preclinical research, the analysis of rodent behavior is necessary to evaluate normal brain processes such as memory formation, as well as disease states like neurodegenerative diseases or affective disorders [1–4]. For centuries, the study of animal behavior was limited by the ability of humans to visually identify, record and interpret relevant behavioral changes in real time [5–9]. Over the last few decades, first computerization and then commercial platforms have stepped in to automate certain measurements, mostly by providing accurate tracking of an animal’s path of motion or nose-point, or by counting mechanical events (beam breaks, lever presses etc.). This has been a game changer for highly constrained experimental testing setups, where a clear behavioral outcome is either expected or acquired over time. Examples include various operant conditioning setups and more complex homecage monitoring systems that can continuously measure predefined daily activities [10–12]. These measures can accumulate large amounts of data over days of continuous recording, yet they do not pose major challenges in terms of data analysis and will not be discussed further in this review. In contrast to these well-automated tests, some of the most popular laboratory tests require ethological assessments, often involving laborious and subjective human labeling. Prime examples are tests that assess the emotional state of an animal (e.g. the open field test or the elevated plus maze, where animals can display a range of exploratory and risk-assessment behaviors), social interaction tests (where two or more conspecifics dynamically interact and display species-specific behaviors), or tests of prey-pursuit (where the actions of the prey and predator quickly influence each other’s behavior output). Amongst these tests, assays of emotionality are usually more tractable, because a single animal is recorded and analyzed. The open field test is an illustrative example, in which the animal is placed in a circular or square enclosure (field). Originally only defecation was recorded as a readout of emotionality and sympathetic nervous system activation [13]. Ambulation was soon added, yet early measurements of ambulation relied on manually counting the number of entries into subdivisions of a grid floor. The importance of recording subtle, ethologically relevant behaviors like rearing, grooming, sniffing or teeth grinding was recognized early on [14, 15], yet the requirement to manually score these behaviors limited the number of parameters that were reported. The advent of computerized tracking through beam-grids or video recordings provided automated measures of distance and time spent in different zones of the open field. This made the open field test one of the most widely used tests in behavior laboratories around the world, providing a quick readout of both locomotor activity and anxiety [16, 17]. While the number of studies using the open field test has exploded over the last few decades, the measurement of ethological behaviors faded into the background. In today’s behavioral neuroscience laboratories, ethological measures in the open field test are largely restricted to scoring a few, well-characterized behaviors (e.g. rearing or grooming), ignoring the rich behavioral repertoire the animal displays during the 10-minute recording session [18]. While automation has thus arguably hurt ethological aspects of behavior, it appears that the advent of machine vision and deep learning tools might now tip the scale in favor of ethology.

### Measuring behavior—the future

Over the last two years, we have witnessed a pose-tracking revolution. Software packages based on deep learning/neural networks allow markerless tracking of multiple, hand-picked body points with astonishing performance [19–24]. From a data perspective, a mouse is no longer only a body center and nose, but has become a complex, three-dimensional skeleton composed of moving vectors. This technology has now been merged with supervised machine learning tools, which enable the detection and quantification of complex, ethologically relevant behavioral sequences with ease and human-like accuracy [25–29]. Additionally, unsupervised machine learning approaches begin to reveal the stunning repertoire of subtle behavioral sequences—often undetectable by humans—that can be recorded with robotic precision in simple testing arenas and in various species ranging from rodents to insects and nematodes [30–34]. This has spawned the field of computational neuroethology, and several excellent reviews have revived the idea to generate a comprehensive behavioral personality profile, an “eth-ome” or “behavi-ome”, in analogy to the genome [35–38]. We direct the interested reader to these reviews, which lay out a thorough general framework for the hopes and challenges big data brings to behavior analysis [35, 36], and summarize the progress made by recent attempts to leverage unsupervised and supervised computational approaches to map the behavioral space of animals [37, 38]. In the present review, we evaluate the various approaches for recognizing and quantifying ethological behaviors using machine learning, and consider the impact of accurate pose estimation algorithms on behavior recognition and quantification. We focus specifically on rodent emotionality, which poses difficult yet tractable challenges for behavioral neuroscience and preclinical work. The findings, limitations, and possible solutions presented here will have important implications for even more ambitious endeavors, such as deconstructing social interactions or analyzing predator-prey dynamics. Based on lessons learned from computational neuroethology over the last few years, we propose practical solutions for how data could be collected, analyzed and shared. These suggestions shall aid the establishment of behavi-omics as a field with defined standards and guidelines akin to genomics.

## From human annotation to machine learning

In the early 2000s, commercial platforms specialized in rodent tracking started offering software packages that enable automated quantification of some ethological behaviors such as grooming or rearing in the open field, or head-dipping in the elevated plus maze [39–41]. Embarrassingly, the current consensus is that these commercial systems still perform poorly when quantifying ethological measures in comparison to human raters [26, 42]. Thus, human scoring has remained the gold standard for a large set of standard laboratory behavior tests, which is extremely laborious and scales with the number of behaviors scored. Annotating every frame in a 1 h video with high confidence was estimated to take 22 person-hours [27]. A study in which experienced raters annotated 13 distinct behaviors reported a time-investment of 1 h per 5 min of annotated video [40], and in our hands annotating a few behaviors in a 10 minute video takes roughly 1 h [26]. In addition, several other human factors limit the reliability and reproducibility of human scoring (see section “The Human is the Limit”). As a consequence, the vast majority of studies using the open field test report only automated measures like distance moved and time spent in the center. Even behaviors that are highly informative about the animal’s emotional state, like unsupported rearing, have been recorded by only a handful of studies over the past several decades [43–45]. Today, supervised machine learning offers a sensitive and reliable solution for automatically tracking complex rodent behaviors.

### Supervised machine learning

#### The basics

**Fig. 1:**
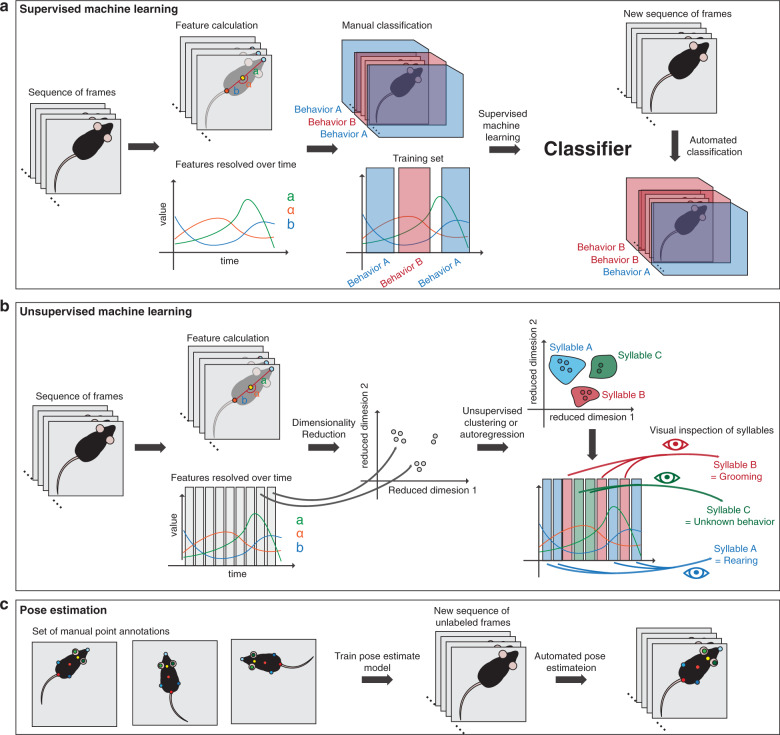
Families of machine learning approaches used in behavioral research. **a** Supervised machine learning methods are used to first train a classifier on manually defined behaviors that then recognizes these based on feature data in new videos. **b** Unsupervised machine learning methods are used to find clusters of similar behavioral syllables without human interaction directly from video data. **c** Pose estimation algorithms track animal body points in videos.

Supervised machine learning requires two preliminary steps: In the first step, video or image data is used to generate a number of features that are used as input for the classifiers (Fig. 1a). Most commonly, animal tracking data from commercial software packages is used to calculate features such as “animal length”, “animal orientation”, “animal speed” or “distance to feeder”. These features are usually manually defined and computed with hand-crafted algorithms, often based on the point-tracking data from the nose and tail base. In this case, “animal length” is defined by the distance between nose and tail base, “animal orientation” by the relative angle of the corresponding vector, “animal speed” by the change of position of the body center over successive frames, and “distance to the feeder” by the distance between nose point and feeder. In the second step, video or image data is manually annotated by human raters, to assign labels (classifications) to individual behaviors. This process is critical, as it establishes the ground truth of what the animal is doing at any given moment in time (see section “The Human is the Limit” for issues related to this process). In the subsequent machine learning step, a mathematical model (classifier) is then trained to recognize the manually annotated classifications. It does this by iteratively optimizing weights/parameters within the mathematical model to increase its accuracy to predict the classification based on the feature data. The classifiers are then validated using a cross-validation data set to estimate its performance on new data. The cross-validation data set is part of the manually annotated data that was not used for training. In the cross-validation process the overall accuracy of the classifier is analyzed when compared to a human annotator. It is important to establish if a classifier only “memorizes” the initial data (good accuracy on training set, but poor accuracy on cross validation set) or if it manages to “generalize” (good accuracy on both sets). There are multiple methods to improve generalization, the most frequently used ones are regularization (punishing the model for using stronger parameter weights) and drop-out (iteratively training the classifier while omitting random parameters in each step). Finally, confusion matrices are often used to visualize pairwise errors of all analyzed behaviors [46]. The confusion matrix not only contains the accuracy of each behavior, but also displays which behaviors are often mistaken for one another between raters.

#### Different supervised approaches

First attempts to use supervised machine learning for automated behavior recognition were made two decades ago using single frames from a side view video recording in combination with feed-forward neural networks [47]. From these single frames three points (nose, center, and tailbase) were set and used to calculate features such as distances between points and angle between vectors. However, no regularization or drop-out was used and the data was split randomly into training and cross validation sets. Consequently, the resulting algorithms performed extremely well on the training set (98%), whereas the cross-validation accuracy dropped to 76%. When different videos were used for both sets the accuracies were further reduced to 84% (training set) and 64% (cross validation set), respectively. Later studies started to incorporate temporal information into the feature space and appropriate machine learning methods to improve generalization. A 2010 study used side-view video recording of mice in their homecage [27] and computed positional features (i.e. distance to feeder), velocity based features (i.e. speed of the bodycenter), and also motion based features that encapsulate temporal information (i.e. filters that can recognize directions of motion). They trained their classifier to recognize eight different behaviors, using an extensive training set comprising 10.6 h of annotated recording. For classification, they used a model based on Support Vector Machines (SVM), which return the most likely behavior at any time, in combination with a Hidden Markov Model (HMM), which takes transition probabilities between behaviors into consideration. They could convincingly show that the “human vs model” confusion matrix was similar to the “human vs human” confusion matrix, demonstrating human-like performance. However, there are some notable limitations to this type of automated recognition. First of all, some of the features rely on positional data (i.e. distance to feeder), which are highly dependent on the set-up. Any change in the environmental configuration would render the models unusable. Additionally, the videos were all recorded from the side, so applying it to a test setup with video from a different angle would require the collection and labeling of a massive new data set. A number of other notable studies have emerged over the last ten years, using different types of features and machine learning methods (see Table 1 for an overview). Most studies calculated features similarly, using hand-crafted algorithms or filters to describe location, appearance and movement of the animal. However, the detail and depth of these features varies a lot, with some studies depending on as few as seven features [48], while others depend on almost 100 [25]. The reported studies (Table 1) indicate that a handful of features can contain enough information to reliably detect some behaviors, and that there might be a marginal increase in performance when including more features. However, it also has to be noted that the inclusion of many manually defined features requires extensive development time and increases the chance of including uninformative/correlated features that can have a negative impact on model accuracy, implying bigger may not always be better [49].

**Table 1:** Studies that implemented automated behavior recognition solutions using supervised machine learning approaches.

Publication	Features	Classifier	Reported Performance	Issues	Available dataset
Rousseau et al. (2000) [47]	3 points	Feed-forward neural network	Sub-human	Overfitting	No
Jhuang et al. (2010) [27]	Location, movement	SVM HMM	Human	Position is cage dependent	Yes
Burgos-Artizzu (2012) [48]	Trajectory, spatio-temporal	Adaboost	Sub-human	Performance	Yes
Kabra et al. (2013) [25]	Appearance, location	Gentleboost	Human	Includes arena dependent features	Yes
Giancardo et al. (2013) [28]	Location, appearance, movement	Temporal random forest	Human	More data needed for some behaviors	Yes
Van Dam et al. (2013) [40]	Appearance, location, movement	GMM	Sub-human	Performance; Company selling the system	No
Hong et al (2015) [76]	Location, appearance, movement	Random decision forest	No human comparison	Relies on different colored animals	No
Lorbach et al. (2018) [41]	Location, movement	GMM	No human comparison	Company selling the system	No
Le et al. (2019) [52]	Spatio-temporal features from 3dCNN (convolutional neural network) 8 × 128 × 128 pixel segments	LSTM	Worse than [27] with same data	Computationally Intensive approach with no increase ìn performance	No
Chaumont et al. (2019) [77]	Intensity histogram, depth histogram (3d camera)	Random decision forest	No human comparison	Requires implanted trackers and 3d cameras	Yes
Nguyen et al. (2019) [54]	Video segments, 224×224 pixels	I3D and R(2 + 1)D	Better than [27] with same data	No transferability assessed	No
Van Dam et al. (2020) [53]	Video segments, 225×225 pixels	3dCNN with multifiber blocks	Better than [40] when data augmentation used	Changes in environment/treatment render it useless	No
Sturman et al. (2020) [26]	Temporally resolved skeleton features from 2D pose estimate	Feed forward neural network	Human, better than commercial solutions	Only few behaviors analysed, transferability not assessed, preprint	Yes
Nilsson et al. (2020) [29]	Temporally resolved skeleton features from 2D pose estimate	Random decision forest	No human comparison	preprint	No, reannotation of [48]

*SVM* support vector machine, *HMM* hidden markov model, *GMM* gaussian mixture model, *LSTM* long short-term memory, *3dCNN* 3 dimensional convolutional neural network, *I3D* two-stream inflated 3dCNN.

#### Tracking multiple animals

In cases where multiple animals are recorded simultaneously (e.g. for social interaction), pre-segmentation enables the separation of the two animals. Pre-segmentation uses established algorithms from image processing practices, such as the watershed algorithm, that can detect borders between objects/animals [50]. Keeping track of the identity of animals is still challenging, especially when animals cross over each other or interact in close proximity), so often different colored animals are used [29, 41, 48]. However, some imaginative methods have demonstrated reliable markerless animal identification for tracking groups of similar-looking animals, such as zebrafish, in set-ups where animal overlap occurs regularly [51]. These methods depend on purely data-driven approaches where a “fingerprint” representation of each animal is created in each frame by using a sequence without any animal overlap. In this case a pixel-pixel pair analysis is used to generate intensity vs distance and contrast vs distance density maps of each animal. After animal crossings, the “fingerprint” with the best match from the previous sequence is found and the animal ID is updated accordingly. These methods can be employed in parallel to automated behavior recognition to additionally resolve animal identity, although it is unclear how well such an approach would perform on mice that are not readily identifiable based on fur patterns. Notably, progress in this area is happening fast. As we are writing this review, a new version of the popular, free pose estimation software DeepLabCut [19, 20] has just been released, capable of tracking multiple animals (including ants, rodents and primates) in simple as well as complex arenas, apparently with astonishing reliability. Given the widespread use of this software package, such an advance will have a major impact, and it is likely that multiple animal tracking will soon be the new standard in the field.

#### New machine learning approaches

In recent years researchers started to adopt emerging technology from video recognition software, employing 3D Convolutional Neural Networks (3D-CNN), which fully prefilter and encode temporal-spatial information on an end-to-end basis. Therefore, no custom algorithms or filters have to be used to define features, these networks rely on video information directly. Results in recent studies have been mixed. In one report [52] the algorithm slightly underperformed compared to the original study using the same data, which had used manually selected features [27]. Another study [53] outperformed an older study using the same data [40]. However, this was only the case when heavy data augmentation was applied and the performance dropped a lot when videos from different set-ups/animals were used. A third study [54] showed a slight increase when using 3D-CNN models pre-trained to recognize human behaviors and retrained with mouse homecage behavior data from a previous study [27]. However, in this study only 12 similar side-view recordings from the same set-up are used, so it cannot be assessed how well it would perform with a different set-up. In parallel to these efforts, supervised machine learning methods have also been developed to automatically recognize mouse social interactions based on ultrasonic vocalization patterns [55], and to recognize emotional states from facial features of mice [56]. It will be exciting to see when all these multi-level analyses converge to construct a multi-sensory behavi-ome.

#### The entry barrier

Most behavioral neuroscience labs do not have extensive knowledge in computer science, programming or deep learning architecture. For these labs, there is a high entry barrier to implement and reproduce the many new machine learning approaches that have been published recently. A notable exception is JAABA [25], a highly polished machine-learning software that is accessible to experimenters without computational backgrounds. It allows the fully integrated annotation and training of new classifiers to recognize new user defined behaviors. It has been independently validated for the detection of grooming behavior in mice [42] and for automatic analysis of fly behavior in a study analyzing over 500 terabyte worth of videos of 400,000 flies [57]. Additionally, there is more good news regarding the lowering of the entry barrier. The recent progress in pose estimation algorithms (Fig. 1c) has resulted in the development of elegant, easy-to-use software packages such as DeepLabCut [19, 20]. These tools enable labs to devise their own point tracking data, which can serve as a high-quality input foundation on which one can define features and train machine learning algorithms. Open-source platforms such as www.openbehavior.com are important drivers for these rapid advancements [58]. For example, user-friendly software interfaces that promise more wide-spread implementation of state-of-the-art algorithms have started to emerge faster than the peer-review process can handle them [29, 59]. One of these tools is Simple Behavioral Analysis (SimBA), a plug-and play pipeline with graphical user interface that uses point-tracking data from DeepLabCut (or other point-tracking solutions) to train machine learning classifiers to detect and quantify behavioral patterns [29]. Currently SimBA focuses on complex social interactions between different-colored strains of rats or mice, but can be used in the simpler open field or homecage setups to score the behaviors of single animals. As noted above, the implementation of robust multiple animal tracking in DeepLabCut will likely galvanize these approaches. It will be extremely interesting to see whether the behavioral research community starts actively implementing these tools, or if it takes commercial all-in-one products to lower the entry barrier sufficiently to truly impact the wider research landscape. If these and other free machine learning tools start being widely adopted, we will learn how well they perform in independent benchmarking comparisons, and how easily the resulting algorithms transfer to the vastly heterogeneous type of video input data generated by labs around the world.

### Unsupervised learning

One of the key weaknesses of using supervised machine learning solutions in behavioral research is the human factor (See section “The Human is the Limit”). In recent years, advances in data-driven unsupervised machine-learning methods (which require no human labeled examples) have transformed the field and are now at the forefront of behavioral innovation [30, 59, 60]. Their inherent strength is that they approach the problem from a data-perspective. Rather than by defining behaviors before tracking them, they observe the entire dataset and look for overrepresented patterns (Fig. 1b). For an excellent description of various computational approaches addressing this issue in different species, we refer the reader to [38]. Here, we focus on the rodent literature, where the most prominent study has employed 3D cameras in combination with autoregressive Hidden Markov Models (AR-HMM) to find sub-second clusters of short behavior sequences, often referred to as syllables or motifs, that act as the smallest building blocks of most behaviors [30]. Upon visual inspection of these syllables by human observers, recognizable behaviors such as “walk,” “pause,” and “low rear” could be identified. The longest sequences that can be found with this approach are in the sub-second range (<500 ms). While it is conceivable that some of these syllables can be interpreted as important behaviors, longer behaviors that are built up from multiple of these syllables in series cannot be sufficiently resolved without any further post-hoc analyses. Other studies involving unsupervised analyses of pose estimation data [59] found longer syllables of up to 2 s length. They further demonstrated that pose estimation data in combination with t-distributed Stochastic Neighbor Embedding (tSNE) and Gaussian Mixture Models (GMM) clustering is sufficient to resolve behavioral syllables. A key element in this study is the dimensionality reduction that has also been incorporated in several earlier studies [31–33, 61]. This process compacts high dimensional data, which can contain many correlated variables each in its own dimension. As a result, a smaller set of variables (=dimensions) emerges that can explain as much of the variability as possible. Thus, this reduced dimension description contains almost as much information as the original description, but is greatly simplified. Dimensionality reduction is not only beneficial for unsupervised methods, but could also be employed for supervised approaches.

## Big data, big problems, small solutions

As we have discussed, many different methods of supervised behavior recognition have been devised to accurately detect complex rodent behaviors. Methods with predefined features are currently fairly well established, whilst end-to-end approaches for supervised learning and unsupervised methods, are very promising but still in their infancy. Some of the major bottlenecks are currently (a) the lack of enough publicly available, well-annotated behavioral datasets to benchmark and compare different algorithms, (b) the lack of a consensus in the field regarding set-ups, camera positioning and optimal features of behavior recognition, and (c) the lack of transferability of classifiers and data. Here, we will discuss these issues and propose solutions.

### The human is the limit

As all supervised machine learning classifiers are trained on data annotated by human raters, these classifiers can only be as good as their human counterparts. Humans are far from perfect at tracking animal behavior, and problems such as high inter- and inter-rater variability, observer bias and observer drift are well-known issues [20, 42, 62–65]. Therefore, the systems trained on the data will never be able to outperform their human instructors. In most cases the ground truth is not perfectly defined and contains a lot of variability. Additionally, there is currently a lack of extensive, well-annotated data-sets, and many studies use older labeled data from previous studies [52–54]. A potential approach could be to create extensive, well-annotated labeling sets, which include only examples where all raters agree with one another, however these would omit difficult cases from the training set and thus limit the sensitivity of the classifier. In this case experimenters could review unclear examples together and try to reach a consensus. However, for cases where reviewers have to draw an arbitrary conclusion because the recording is unclear, the ground truth cannot be established. Additionally, our human sensory modalities likely limit our ability to recognize subtle behavioral motifs that might be of crucial importance to the animal’s repertoire of behavioral expressiveness, similar to our inability to hear sounds in the ultrasonic range. While unsupervised analyses promise a way around human bias, they are also ultimately constrained by human interpretation. Unsupervised approaches discover behavioral categories based on their natural statistics, which makes these data-driven approaches particularly appealing. Once the unsupervised approach reports a list of behavioral categories that have been altered by an experimental manipulation, it again requires human intuition to derive a meaningful behavioral interpretation. Thus, a human experimenter has to visually inspect examples of altered categories and connect them to known behavioral readouts. This could be especially tricky for short behavioral syllables that cannot be connected to any known readout. In this case, what do we do with an altered syllable that cannot be linked to any previous observations in the literature? How can we ensure that they are important readouts and not simply correlated observations due to a simpler explanation, such as reduced motility/activity, which can be recorded with a simple center of mass tracking? This is connected to the issue of time segmentation, the difficulty of deciding at which point syllables should be distinguished as separate entities, or whether multiple syllables should be clustered into one behavior. Further, should similar behaviors be clustered or separated (e.g. low vs high rears)? If these two indicate the same phenotype, analyzing them independently would not only increase variability, but also the number of behavioral variables tested (thus hurting statistical power, see section “The Multiple Testing Problem”). Despite these theoretical difficulties, first attempts at deconstructing open field behavior using an unsupervised approach suggest that well-known behavior categories emerge, like rearing and grooming, and that finer nuances in grooming can be detected (face groom, head-groom, body licking, paw licking), some of which carry biologically relevant information and respond selectively to brain circuit interventions [30, 34, 59].

### The multiple testing problem

Supervised approaches can track large numbers of complex behaviors in a given test, and unsupervised methods may identify many new unexpected behavioral categories (e.g. several sub-categories of grooming or transition patterns between behaviors). With an increasing number of dependent variables, multiple testing considerations become extremely important to prevent a high degree of false positives (type-I errors), yet these considerations are most often ignored [66–68]. If we evaluate five independent behaviors in the open field test, and we find one significant difference between two groups of mice (*p* = 0.05), the chance of this being a false discovery is not 5% anymore, but ~23% (1–0.95^5^). Mathematical tools have to be applied to correct for multiple testing, which can be rather stringent, such as the Bonferroni adjustment, thus greatly reducing the power of the test. Many more animals would have to be used per group to reveal statistically significant differences, which violates guidelines to reduce animal numbers wherever possible [69, 70]. An false discovery rate (FDR) correction [71] is much better at reducing both type-I and type-II errors by analyzing the p-value distribution of multiple observations, yet this type of analysis makes the assumption that all tests are independent. For many behavioral read-outs this is clearly not the case as many behaviors correlate strongly (e.g. distance and velocity). An appropriate multiple testing correction, such as the refined Benjamini-Yekutieli procedure, assumes that tested variables can correlate [72]. However, even the correct statistical approach does not solve the key issue: the more dependent variables we analyze, the more power we need to detect differences. This argues for carefully deciding which behaviors to analyze a priori when using supervised models, or to limit the number of detected clusters when employing unsupervised methods.

### Data and model transferability

How well can a classifier recognize behaviors in a new data space? For example, can a classifier trained to recognize grooming in the open field test also recognize grooming in the elevated plus maze or in the homecage (in the same lab)? Can a classifier trained on how to recognize behaviors in the open field test in one lab recognize the same behaviors equally well in an open field test that is slightly different (in terms of lighting, size, orientation, camera distance etc.) in a different lab? And how does performance change when testing animals of a different sex, age, or strain? This concept of “model transferability” is often ignored, but might be one of the most important characteristics. The reason the open field test is so popular, and most studies report only distance travelled and time in center, is because it can be easily automated and standardized across labs. If a model has poor transferability, anyone who wants to establish a similar workflow in their lab will face a large investment in terms of labeling new data and then training and evaluating new classifiers. This process is not only costly, but also limited to researchers with a sufficiently strong computational background.

For models that use manually selected features, several characteristics determine transferability. For example, are any of the features position or rotation dependent? Features such as “distance to feeder” or orientation within the arena will not allow for easy transfer of the models into new contexts, since these features might either be non-existent (no feeder), or have a different distribution in the new context (orientation). Most studies cited here are subject to these problems. Although high hopes have been placed in end-to-end systems, recent studies have shown that they seem to be even less transferable [53]. One problem with such end-to-end approaches could be that the “data-space” is much larger than when only a few selected features are considered (see section “Data and model transferability”). Classifiers have difficulties in generalizing when data is presented that looks different to previously encountered data (e.g. different illumination, different lens effects, different-looking rodent strains). Despite these issues, the benefits of a transferable end-to-end system would be immense, because it would circumvent many of the biases of manual feature selection. An ambitious solution could be to create an extensive data-set that contains annotated videos of numerous different animals (mouse strains, different ages, sexes, sizes) in many different set-ups, recorded with different cameras/lenses, zoom levels, at variable lighting conditions and with heavy data augmentation. However, the success of such a model is hard to predict and the time-investment would require a multi-center study with large investment from many research groups.

Transferability also appears to be a major issue of unsupervised methods. A pioneering study that directly modelled pixel data found that their approach strongly depended on the size and shape of the mouse [30]. The number and type of syllables observed depends on these and other variable conditions. How a syllable observed in one setting differs to the same syllable in a second setting is unclear. How can we ensure that the same “cutoff” point between similar syllables is found by the algorithm each time? In addition, if data of multiple experiments from different set-ups are included in an unsupervised analysis, how do we prevent the algorithm from considering the same behavior in two set-ups as separate syllables (e.g. “grooming experiment 1” and “grooming experiment 2”)? Even If more data from the same setup is used, unsupervised methods will necessarily increase the number of discovered syllables [30].

### One solution to the transferability problem

Powerful new pose estimation tools now enable the necessary 3D point-tracking of multiple body points with very high accuracy [19–23, 73]. They are easy to implement and adjust, creating a new model to track multiple points on a freely moving mouse in a new environment takes roughly 12 h [26], and does not require advanced computational expertise. Therefore, rather than generating a massive dataset of annotated behavior videos to train robust, transferrable end-to-end solutions, we propose to generate a transferable skeleton representation of the animal from raw video data. The major advantage of this approach is that the resulting three-dimensional skeleton representations are completely independent of the animal’s position and orientation, and also of environmental factors such as illumination and background. The skeleton contains vectors constructed from point-pairs. These vectors can be used to either generate a number of features that are fed into supervised machine learning algorithms [26], or these features can directly form the input for unsupervised clustering, as was recently demonstrated [59]. Absolute distance would be the first class of features, angles between vector-vector pairs and the angle to the Z-plane (=ground level) can be included as second and third classes. The advantage is, that angles are scale-less by nature, whereas distances can be scaled linearly for data augmentation or efficiently normalized (see Box 2). This could be the most transferable description of a freely behaving animal, and should be able to be analysed with classifiers trained on a completely different dataset. Additionally, in combination with arena data (e.g. polygons, points of interest etc.), these point-tracking data retain any important information for animal behaviors that are dependent on specific set-ups.

**Fig. 2:**
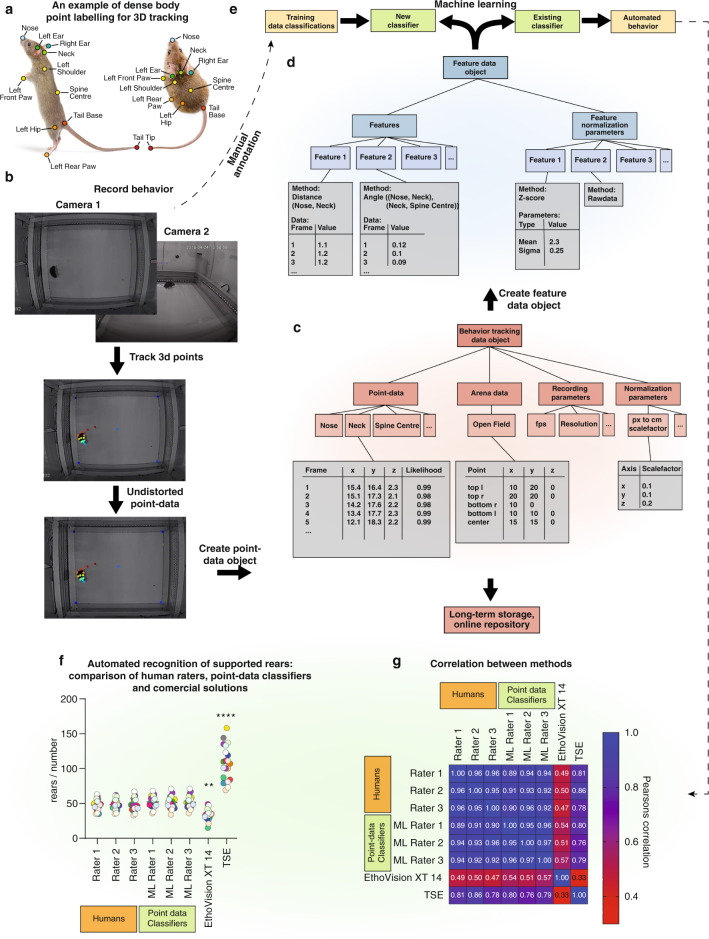
Proposed workflow for high-fidelity, transferable behavior recording. **a** A high-fidelity point-set is selected that retains most of the animal information for the least storage space required. **b** Behavioral tests are recorded from multiple perspectives with synchronized cameras. Pose estimation algorithms such as DeepLabCut are used to track the defined points. Undistortion is applied either to the videos directly or to the data. **c** Tracked point data is used to create a behavior tracking data object that contains all essential information about the behavioral test that can be used for any post-hoc analysis. This object is used for long-term storage in online repositories. **d** Behavior tracking data objects can be used to create a feature data object that contains all features that are important to recognize a selected behavior. Setup-specific normalization factors are contained within the feature object to allow easy transferability. **e** Feature objects are used in combination with existing classifiers to automatically track behaviors, or a new classifier can be trained in combination with manually annotated training data. **f** Example data comparing the proposed workflow to commercial solutions (Ethovision XT 14, TSE Systems) and humans. Supported rearing behavior is recognized with human accuracy when using features generated from 2D (top view) point-tracking data (adapted from ref. [26]. **g** Correlation between three human raters, the machine learning classifiers, and the commercial systems from the same study.

Although a 3D skeletal representation is intriguing, it also comes with several caveats that need to be addressed. First, data has to be presented in an undistorted format (Box 1) and has to be sufficiently standardized (Box 2). Further, the set of points included in the tracking has to be sensible and retain as much information as possible. For example, tracking the nose, headcenter, headbase, bodycenter and tailbase enable a fairly accurate in silico modeling of the spine. This can pick up behavioral syllables such as head direction or body curvature through angles between spine vectors, and stretching/hunching through distances between spine points. If we move from 2D tracking to 3D tracking, further syllables such as rearing can be determined fairly accurately through observation of angles between spine vectors and the z-plane. However, the perfect set of tracked body points (if such a universal description exists for rodents) needs further investigation. Since features generated from point-data do not have to include all points (Fig. 2d, e), tracking more points than required is not detrimental to machine learning, but requires more human labelling [26]. A sensible approach for point optimization would be to start with a broad selection of body points, then successively drop points and assess the accuracies of the resulting classifiers. An overarching analysis could reveal which points are the most important for the largest groups of behaviors. Automated types of feature selection (Guyon 2003) could be employed on the full point-set, and points associated with most redundant/uninformative features dropped. For ease of transferability, the point data should be stored and shared with a representative and unambiguous description of the point set, and the criteria used to set them. Here we propose a set of points that contains the nose, headcenter, headbase, bodycenter and tailbase (the spine points), as well as the ears, hips, shoulders and paws (see Fig. 2a). These, in combination, should enable a description of the most important axes of movement and have proven efficient for recognizing and dissociating supported and unsupported rears, two very similar behaviors in the open field test [26]. Only six body points were sufficient to dissociate different forms of grooming activity (which are difficult to score for humans) in a recent effort to combine point-tracking with unsupervised behavior recognition in the open field [59].

Box 1 Dealing with lens distortions Coordinates from point-tracking algorithms depend on the x- and y-pixels in the video images. Many lenses have distortions, sometimes clearly visible, sometimes less noticeable but still present. Distortions are problematic because they alter lengths and directions of vectors, especially in the periphery (see Fig. 2b), therefore slightly altering the pose-description. From a data-perspective, the same behavior looks different in the periphery than in the center of the field. Distorted videos can still be used for training classifiers, but much more training data is required to reach a high accuracy. Long-focus lenses solve this issue, as they have almost negligible lens distortions. However, they have to be placed much further away, making setups more cumbersome. Wide angle lenses can be placed much closer but are subject to very strong distortions. For wide angle lenses software solutions can undistort the videos before they are used as input for point tracking (Fig. 2b). Alternatively, the distortion can be removed from the data directly. This requires one or multiple calibration frames to be imaged with the same set-up. Sophisticated point-tracking methods such as deeplabcut fortunately incorporate solutions for undistortion [19].

Box 2 Normalization and standardization of features generated from point-data To ensure transferability of models, features used to train classifiers must be independent of the setup. If the feature “body length” is calculated based on point tracking data (e.g. distance from nose to tail), the resulting length will depend on the camera location relative to the animal. This feature cannot be readily transferred and needs to be normalized or standardized. In contrast, features such as angles (in rad) or true/false checks are perfectly transferable. There are two commonly used approaches for machine learning, min-max normalization and z-score standardization. Min-max normalization linearly transforms data so they fall into the range [0,1], setting the maximum value to 1 and the minimum value to 0. For comparison across datasets this can be dangerous, since outliers will define the range and have a strong effect on the mean value of the data of a given feature. Z-score standardization rescales based on a normal distribution, where 0 is set to the mean value of a given feature and one standard deviation is rescaled to ±1. There are two advantages to standardized data. First, it is more comparable across set-ups, since it will correct any set-up specific scaling alterations (i.e a zoom factor or a mouse size factor). Second, it optimizes the training speed of most types of classifiers that use iterative processes such as gradient descent, since the upgrade function can use the same learning rate for all features. Standardization parameters (mean value of features and standard deviation of features) should be determined for each set-up across multiple normally behaving wild-type mice and should then be included into the feature-data object (Fig. 2d) to enable appropriate re-scaling. In addition to correcting for spatial differences, temporal differences between setups have to be corrected for as well. Most accurate classifiers will incorporate temporal information (i.e. how the pose changes over multiple frames), but the sequence of a 15 fps recording will look different to a classifier than a 25 fps recording. An easy solution is to linearly interpolate data acquired with different fps to match the classifiers trained for a specific fps. For slower recordings, additional data has to be generated where tracked points of inferred frames are linearly placed between the positions in recorded frames, whereas for faster recordings data has to be removed. Inevitably, different research questions require different frame rates (e.g. whisker recording vs. locomotor tracking). In order to pick up a signal with a set frequency, the sampling frequency should be at least twice as fast, as stated in the Nyquist–Shannon sampling theorem [78], however for complex behaviors having at least ten data points per occurrence is preferred. Therefore, it reasonable to believe that there should potentially be more than one standard framerate, one for standard entire body rodent behavior (~25–30fps, assuming behaviors are longer than 500 ms) and one for fast behaviors, such as sniffing or whisking ~200fps for ~15 Hz).

### Data storage, memory and computational requirements

#### Data storage

Despite an exponential drop in the cost of storage media, long-term storage of raw video data can still be a challenging issue. If raw video data is kept at all, tape-based storage is the most economical solution, rendering the original video recording inaccessible to other researchers. Moving to high resolution, high frame rate and multiple angle recordings further increases memory demands. A full day (24 h) worth of 3D recording using three camera angles would require almost 45GB of data storage (full HD with mp4 compression). Storing these amounts of data is no problem nowadays, but if such data streams are collected daily or weekly, storage will become challenging for most labs (published data needs to be archived for many years). Our proposed approach of using point-data for tracking, training and distribution (see section “One solution to the transferability problem”), solves this issue. Data size grows linearly with the number of points tracked and remains in a manageable magnitude. One day of continuous 3D tracking would reach 35MB per tracked point (assuming 25 fps, 4 values per point: x,y,z and likelihood, all in single precision). Therefore, 20 points would result in a 3D data description of 700 MB per day of continuous recording. Adding more points is cheap and enables a fairly complex time-resolved reconstruction of an animal in 3D.

#### Working memory

All methods that use feature selection (such as various pose estimation approaches) will immensely reduce the amount of data that needs to be loaded into working memory. A highly compressed, low resolution video with uniform background requires ~2 KB per frame. If a video is pre-processed with an algorithm that determines 20 features, all numeric single precision (4 bytes per feature), the memory of the feature data per frame is 0.1KB. Therefore, using features, a whole day of recording data at 25 fps can be represented with ~200 MBs (a size even low-end systems can easily load entirely). For end-to-end systems this number explodes, as these models do not work with compressed data directly, so videos have to be decompressed before any computation is performed. Frequently, the animal part of the videos is re-segmented into a more manageable size, for example 224 × 224 pixels [53]. After resegmentation, a black scale representation with an intensity depth of 256 levels (=1 byte per pixel) now requires ~50 KB of working memory per frame. A day’s worth of recording (24 h) thus requires ~100 GB for data representation. On top of that, end-to-end systems have been shown to work best with data augmentation [53]. Therefore, this number could easily reach hundreds of GBs. Expensive systems with immense amounts of accessible working memory are necessary to efficiently handle and train models with this amount of data.

#### Computation

Computationally, the bottleneck for most supervised and unsupervised methods is training the classifiers or clustering the data. The computational load depends on other variables such as learning rate, number of iterations/epochs and more, which themselves depend on the data depth, complexity and variability. It is thus hard to estimate the computational need of each model, but obviously larger data sizes are accompanied by greatly increased computational needs during the training phase. Therefore, end-to-end models, which use raw video data as input, are the most demanding solutions [74] and often require access to expensive systems such as multi-GPU desktop computers or cloud services to efficiently train. Whereas models with more manageable feature dimensions can be trained on a standard desktop computer.

#### The need for consortia

New technologies that improve behavior tracking are emerging extremely rapidly, yet without standardized practices for data acquisition, processing and sharing. What is lacking are (1) quality standards and data formats for publicly sharing behavioral data, (2) the necessary online repositories, and (3) guidelines that require authors to deposit published data online. In comparison, the field of genomics has resolved these issues through consortia. There are clear rules how genome/transcriptome sequencing data is recorded, saved and distributed in a transparent way that allows other researchers to access unprocessed, raw data and reanalyse them with their own methods and meta analyses. The main idea was to settle on a raw data format that is data independent, i.e. it does not depend on specific genome or transcriptome assembly. In transcriptomics, ‘.fastq’ files are saved. They contain every individual sequencing read that was measured, and includes corresponding quality metrics. Whenever a study is published, these unprocessed files are deposited in an online repository, such as the gene expression omnibus, where they cannot be altered or removed anymore and are accessible for anyone [75]. These standards are enforced by journals and reviewers. For behavior, there needs to be a similar system where a data-independent measurement produces raw data that is then shared efficiently and can be reanalyzed by a different research group with little effort. While the smallest unit in genetics are individual nucleotides, the smallest units of behavior remain unknown. There are good arguments for uploading raw videos, which should become a standard in the field, yet there is one key issue with uploading videos alone. Videos vary dramatically in terms of quality, and it makes no sense to standardize video recordings as they depend on experimental setups. Videos will have different resolution, frame rate, angles, brightness etc., which makes them data-dependent and thus difficult to analyze with general data processing pipelines. Further, single videos are not very informative, since they are biased by perspective. Uploading videos from multiple perspectives that enable 3d reconstruction of movement could solve this issue, yet would still not enable others to feed these data into standardized pipelines. A solution based on point-tracking data could be to share processed 3d pose estimations of a high-quality reference point-set. These would contain time-resolved pose data of individual animals and would enable fast and efficient meta analyses with the advantage of a strong data compression and simplification at the cost of being not completely data independent. Such a stipulatory data format would also address the lack of large, well-annotated and standardized behavior data-sets, as it would enable researchers to collaborate at producing well polished data-sets that contain annotated examples from many different set-ups, mouse lines and mouse models. This would help develop better supervised and unsupervised algorithms, which depend

on large and well-annotated datasets to be trained and validated. Benchmarking different machine learning models would become easier, and we could test how well models transfer between labs or setups. Finally, as easy-to-use machine learning tools with intuitive user interfaces for behavior analysis are emerging [25, 29, 59], combining them with a standardized data format would enable a seamless integration of emerging technologies into the labs of behavioral researchers.

## Future directions and clinical applications

Behavior assessment is one of the cornerstones of preclinical research, as changes in animal behavior are the main readout before new compounds can enter clinical trials. Therefore, advancements in our ability to reliably detect and quantify ethograms in laboratory rodents and other species will ultimately impact human health. The field of behavioral research is currently experiencing the rapid innovation and implementation of new technologies. These advances bear incredible potential, not only to enable large scale and fully automated analyses, but also to increase the quality of the data and the depth of the information that can be extracted from a single behavioral experiment (see Box 3). Nonetheless, the field faces major challenges: First, the large number of novel variables makes it harder to interpret the outcome of an experiment and requires more sophisticated statistical tools for analysis. Second, these novel approaches generate large amounts of complex data, the analysis of which require high-performance computing resources and advanced knowledge in computer science and deep learning architecture. Third, as these approaches begin to be implemented by different groups, many new issues emerge: repositories for sharing raw behavior data—and the necessary consortia and guidelines—are not available, the lack of standardization leads to issues in transferability (between different laboratories, tests, or setups), and the absence of benchmarking datasets makes quality assessment difficult. Finally, whilst a few research groups push the boundaries of what is technically feasible, the entry barrier to deploy these tools is still too high for the large majority of research labs, which generates two parallel worlds of behavioral analysis. Going forward, behavioral scientists will have to adapt new skills to implement new technologies, and we urgently need to form consortia to establish timely standards and guidelines that enable data sharing and foster reproducibility. As a group, we need to learn from fields like genomics to unlock the full potential of behavi-omics.

Box 3 Practical benefits of machine learning approaches for quantifying behavior Machine learning approaches have already revolutionized the detection, quantification and analysis of animal behavior. We elaborate on this throughout the review, while also emphasizing current limitations and the challenges ahead. Here, we summarize the advantages of machine learning by categorizing its contributions into automation and exploration.

**Automation**. Supervised machine learning approaches provide a powerful means to automate laborious tasks, thus saving valuable time for researchers and standardizing analysis. Typically, a research lab can make a one-off investment by establishing a detailed annotation of behavior videos for a given task, and then all future data can be analyzed using the same algorithm. This not only saves time, but allows for important standardization as it avoids differences between raters to impact the results. While transferability of algorithms between labs is currently still an issue (see section “Data and model transferability”), it will certainly soon be possible to transfer fully trained (ideally community-curated) classifiers between labs. This would eliminate both the initial work investment, and between-lab variation that arises from different raters and from subtle differences between definitions of behaviors. Prominent examples include the successful quantification of (1) unsupported vs supported rears [26] and (2) grooming activity in the mouse open field test [42], 3) complex social interactions between two rodents [29], and (4) multiple fly behaviors to successfully correlate them with the activation of thousands of genetically targeted neuron populations [57].

**Exploration**. The unique advantage of unsupervised approaches is the ability to identify previously unrecognized behaviors or behavioral syllables, and to afford an unbiased “exploration” of datasets based on statistical clusters that explain between-group variability. Prime examples are the identification of short behavioral syllables that form building blocks of rodent behavior and can be correlated with activity in specific neural pathways [30, 34], and the identification of different subtypes of grooming behavior in the open field test, which are difficult for humans to accurately detect [59].

**(Un)constrained behavior**. So far machine learning approaches have mainly been employed in unconstrained tests where mice can freely express behaviors (e.g. open field, elevated plus maze, free social exploration). However, these approaches could also be used in more constrained “task-based” designs, where mice are e.g. trained to press levers or use memory cues to explore specific places or objects in a maze. While the primary outcome measures will depend on straight-forward tracking (e.g. number of lever presses or time spent investigating a specific object), additional ethological variables could easily be recorded and analyzed using (un)supervised approaches. For example, rather than only measuring time investigating objects in the novel object exploration task, rears and stretch-attends toward the novel object could yield measures of risk-assessment and approach-avoidance conflict, in addition to an isolated measure of memory strength. For a detailed discussion regarding the potential of big data in bridging the gap between constrained and unconstrained behavior testing, we refer the reader to an excellent perspective article by Gomez-Marin et al. [35].

## Funding and disclosure

The lab of JB is currently funded by the ETH Zurich, SNSF Project Grant 310030_172889/1, ETH Research Grant ETH-20 19–1, Kurt und Senta Herrmann-Stiftung, and Botnar Research Center for Child Health. The authors declare no competing interests.
